# Supplementary material for: Wheat Ms2 encodes for an orphan protein that confers male sterility in grass species
Source: Nat Commun. 2017 Apr 28;8:15121. doi: 10.1038/ncomms15121 (PMC5414350; doi:10.1038/ncomms15121)
Supplement: Supplementary Information — Supplementary Figures, Supplementary Tables and Supplementary References [file ncomms15121-s1.pdf]

## Supplementary Figures

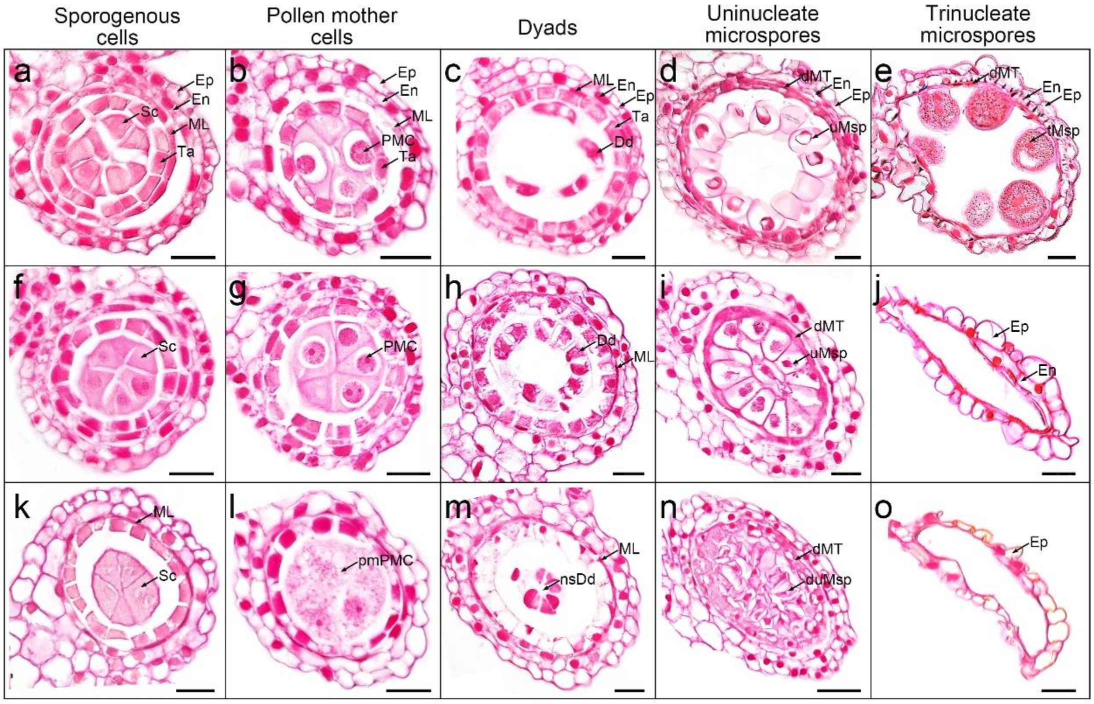


**Supplementary Figure 1. Disruption of microsporogenesis in Taigu lines.** Transverse sections of anthers were prepared from wild-type male-fertile (MF) wheat (**a-e**) and male-sterile (MS) Taigu lines (**f-o**) at the five stages (marked on top of the columns). The plants are MF lines of XY6 (a,d,e) and CS (b,c), and MS lines of $CS_{RMs2}$ (f-h,l,m,o) and $XY6_{Ms2}$ (i-k,n). The Taigu lines have various cellular disorders: 1) premeiotic degeneration of the middle layer in k, 2) plasmodial mass of pollen mother cells in l, 3) non-separating dyads in m, 4) a common degeneration of uninucleate microspores in n, and 5) a 'pollen-less type' sac[1] in j and o. Abbreviations: Ep, epidermis; En, endothecium; ML, middle layer; Ta, tapetum; PMC, pollen mother cell; Sc, sporogenous cell; Dd, dyads; nsDd, non-separating dyads; uMsp, uninucleate microspore, tMsp, trinucleate microspore; dMT, degenerated middle layer and tapetum; duMsp, degenerated uninucleate microspore; pmPMC, plasmodial mass of pollen mother cells. Scale bars = 50µm.

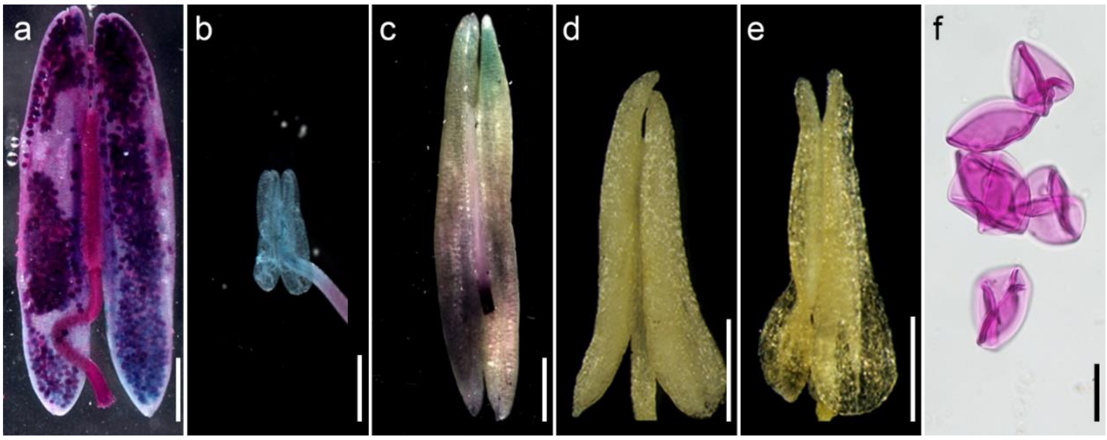


**Supplementary Figure 2. Spectrum of morphology of Taigu anthers.** (**a**) An anther from a wild-type male-fertile (MF) wheat plant stained by Alexander's method. (**b, c**) Anthers from male-sterile (MS) plants from popA-2 (XY6$_{Ms2}$/2*SW41) stained by Alexander's method with a diminutive anther (b) and a more normally-sized anther but without viable pollen (c). (**d, e**) Late MS florets from a MS plant in the *RMs2*-based recurrent selection population had more normally-sized anthers but the pollen (**f**), stained by Alexander's method, was non-viable. The anthers of LM15$_{RMs2}$ and other Taigu lines did not typically bear any pollen. Late flowers in Taigu lines occasionally bore more normally-sized anthers[2]; however, the Taigu anthers were typically thin and rigid; any pollen that was produced was non-viable. Scale bars = 500 µm (a-e) and 25 µm (f).

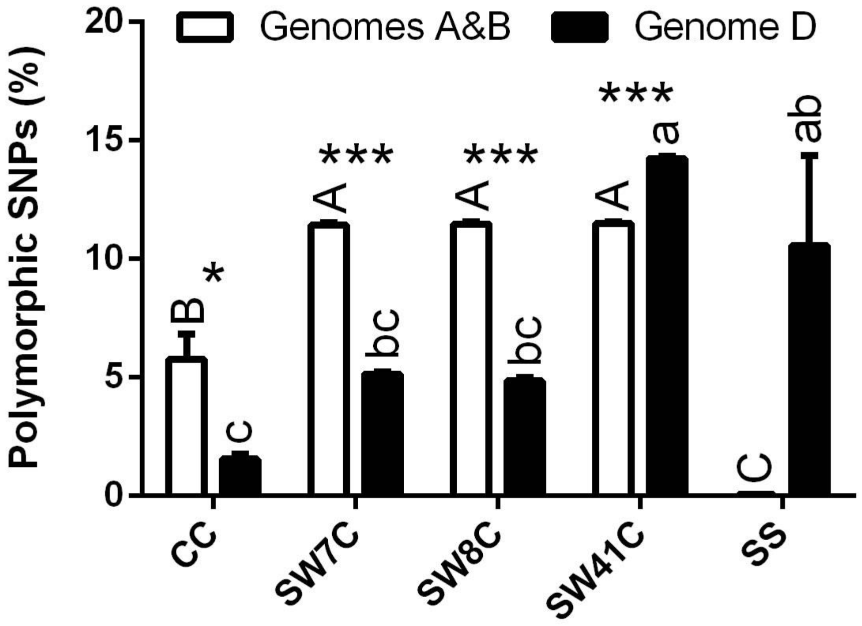


**Supplementary Figure 3. DNA polymorphisms amongst parental lines that were used to map *Ms2*.** DNA polymorphisms were detected using the wheat 90K iSelect SNP assay[3]. Comparisons were made within common wheat (CC), and the synthetic hexaploid wheat (SS), and between the synthetic hexaploid wheat and common wheat (e.g. SW7C). Taigu lines are in the common wheat lineage. For each *X*-axis group, a mean polymorphism rate was obtained by averaging the within group comparisons (n=3). The error bar is one standard error of the mean (s.e.m.). Significant differences between genotypes in the percentage of SNPs were determined by a Tukey-Kramer HSD ($\alpha$ =0.05, capital letters for the AB genomes and small letters for the D genomes). Analysis of significant differences in the percentage of SNPs for each genotype in the AB versus D genomes was done with paired *t*-tests (asterisks: * $P$<0.05, *** $P$<0.001).

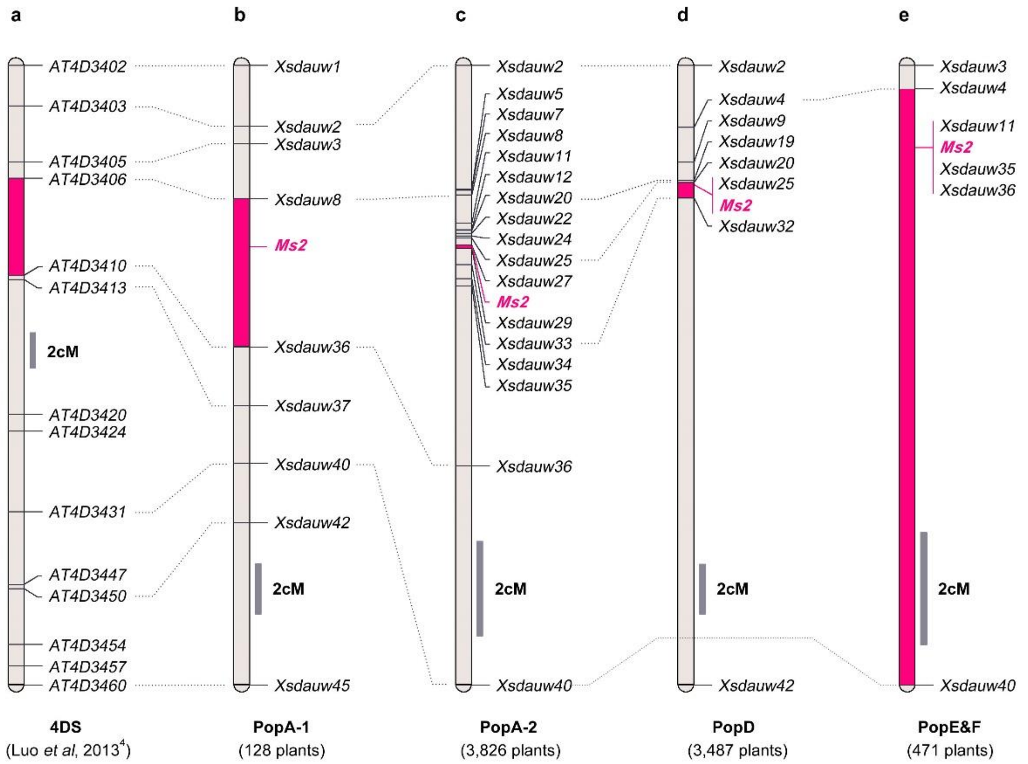


**Supplementary Figure 4. Mapping of *Ms2* in Taigu lines.** (**a**) The 4DS linkage map of *Ae. tauschii*[4]. (**b**) A preliminary map from popA-1 ($XY_{Ms2}$/2*SW41) using 43 plants for the linkage map and 85 plants for validating the *Xsdauw8-Xsdauw36* interval. (**c**) Fine map of the *Ms2* region based on popA-2 ($XY_{Ms2}$/2*SW41, 3,826 plants). (**d**) Fine map of the *Ms2* region based on popD ($XY_{Ms2}$/2*SW7, 3,487 plants). (**e**) An integrated map from popE ($LM_{RMs2}$/2*SW41, 235 plants) and popF ($CS_{RMs2}$/2*SW41, 236 plants). *Xsdauw32* and *Xsdauw33* are derived from the same region, where the *Xsdauw33* range is nested within *Xsdauw32*. The pink region represents the *Ms2* interval. The *Xsdauw4/Xsdauw5-Xsdauw40* intervals are genetically comparable, about 10.5 to 10.8 cM, in both *Ms2*- and *RMs2*-based populations (popA-2, popE, and popF). The *Xsdauw11-Xsdauw36* interval is about 5.1 cM and spans *Ms2* in popA-2, but both markers were completely linked to *Ms2* in the *RMs2*-based populations popE and popF.

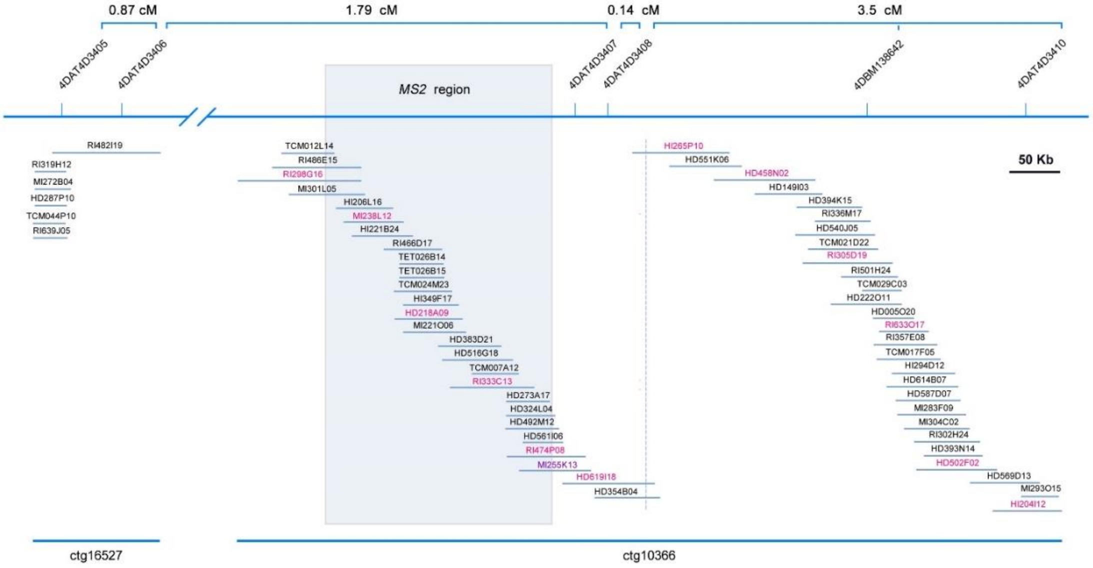


**Supplementary Figure 5. Physical contigs of the *ms2* region in *Ae. tauschii*.** Genetic distances and physical contigs are from a previous report[4]. Overlapping BACs in pink or purple were sequenced to span the *Ms2* interval. BACs in black were not sequenced. The *MS2* region is highlighted by gray shading. A previous study revealed a complete linkage between 4DBM138642 and 4DAT4D3410[4].

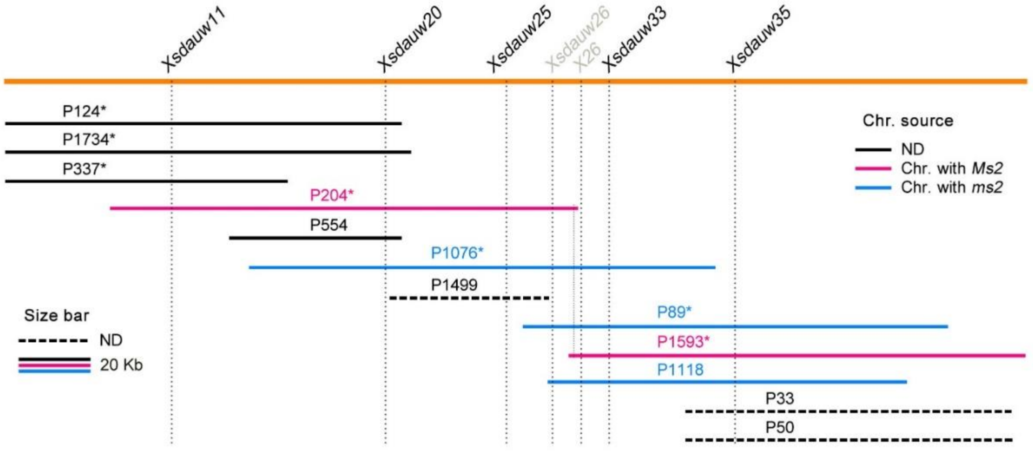


**Supplementary Figure 6. Physical map of the *Ms2* region in LM15$_{RMs2}$.** Five markers in black were used to screen the LM15$_{RMs2}$ BAC library; two markers in gray (*Xsdauw26* and *X26*) assisted in differentiating between the *ms2* and *Ms2* associated chromosomes. P204 and P1593 shared 1,212 bases and formed a complete physical map of the *Xsdauw11-Xsdauw35* interval in the *Ms2*-associated chromosome (BACs in pink). P89 and P1076 formed a complete physical map of the *Xsdauw20-Xsdauw35* interval in the *ms2*-associated chromosome (BACs in blue). The chromosome source was not determined (ND) for seven clones (BACs in black).

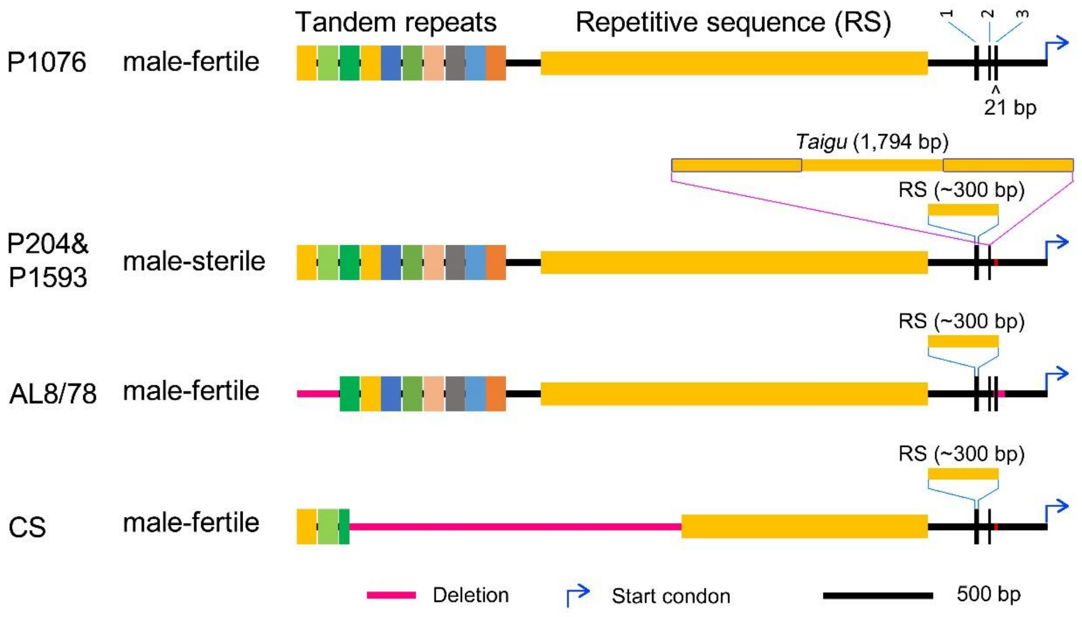


**Supplementary Figure 7. Major structural variations in the *PG5* promoter region.** A 3,502-bp region was analyzed. The first 2,894-bp contains three to ten tandem repeats and then a large repetitive sequence (RS). The remaining 608bp is not repetitive overall, and has three structural variations: 1) a RS insertion in all entries except for MF P1076, 2) a *Taigu* retroelement insertion in MS P204 and P1593, and 3) a small fragment deletion in all entries except for P1076. Here, P204 and P1593 have the only male-sterility allele. All other lines (AL8/78, CS, and LM15) are male-fertile. The base numbers are based on the $PG5_{P1076F}$ (= LM15).

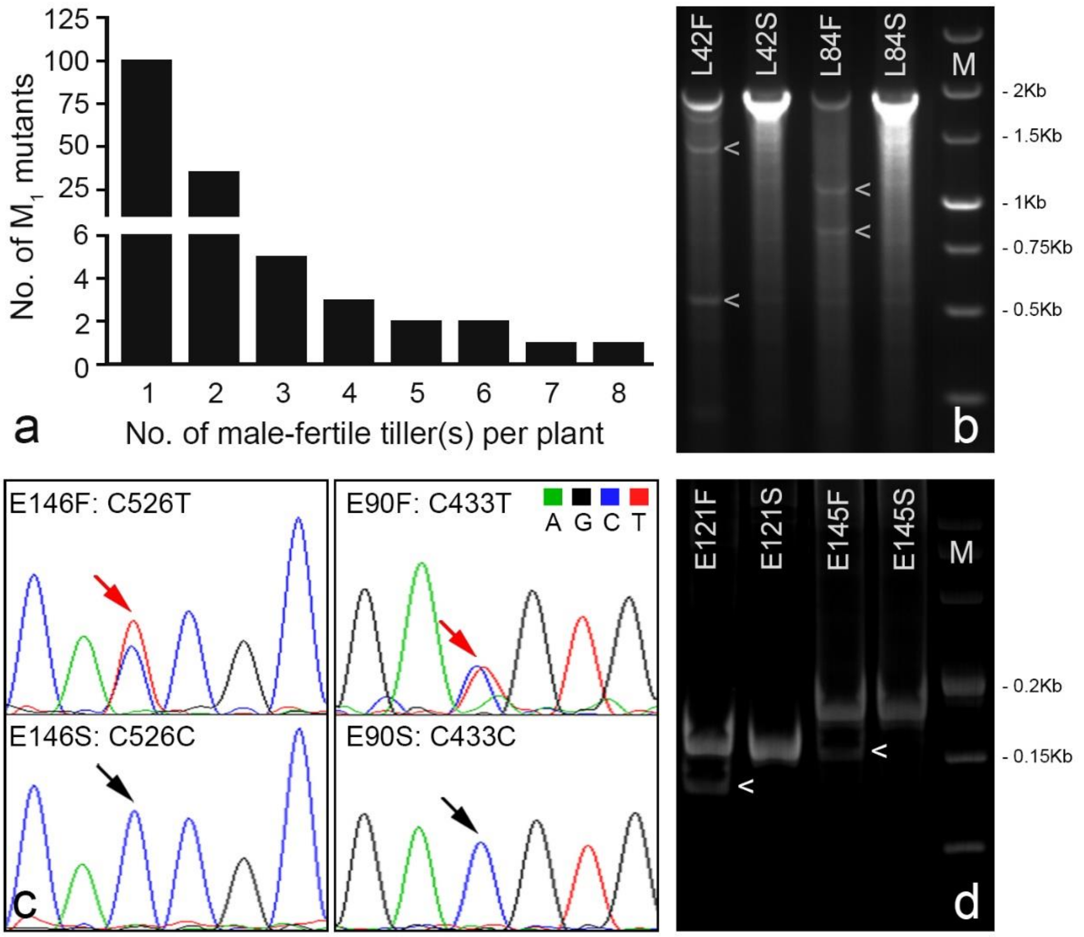


**Supplementary Figure 8. EMS-induced mutagenesis of the *PG5*$_{P1593S}$ gene.** (**a**) Distribution of male-fertile (MF) tillers in the CS$_{RMs2}$ subset. (**b**) TILLING screening (P133/P134, 1,937 bp) of the segregating tillers of the same $M_1$ plants (S: sterile tiller; F: fertile tiller). (**c**) Sequence chromatograms of selected mutations. (**d**) Marker-assisted validation of the identified mutations (T1 and T2 markers for the C158T and G437A mutations in E121 and E145, respectively; Supplementary Data 4 and 5). Arrows and arrow heads highlight the *Cel*I-digested band (b), the base change (c), and the *Hpy*188III-digested band (d). M, the DS5000 DNA Ladder (Dongsheng, Guangzhou, China; in b) or the DM500 DNA Ladder (CWBIO, Beijing, China; in d).

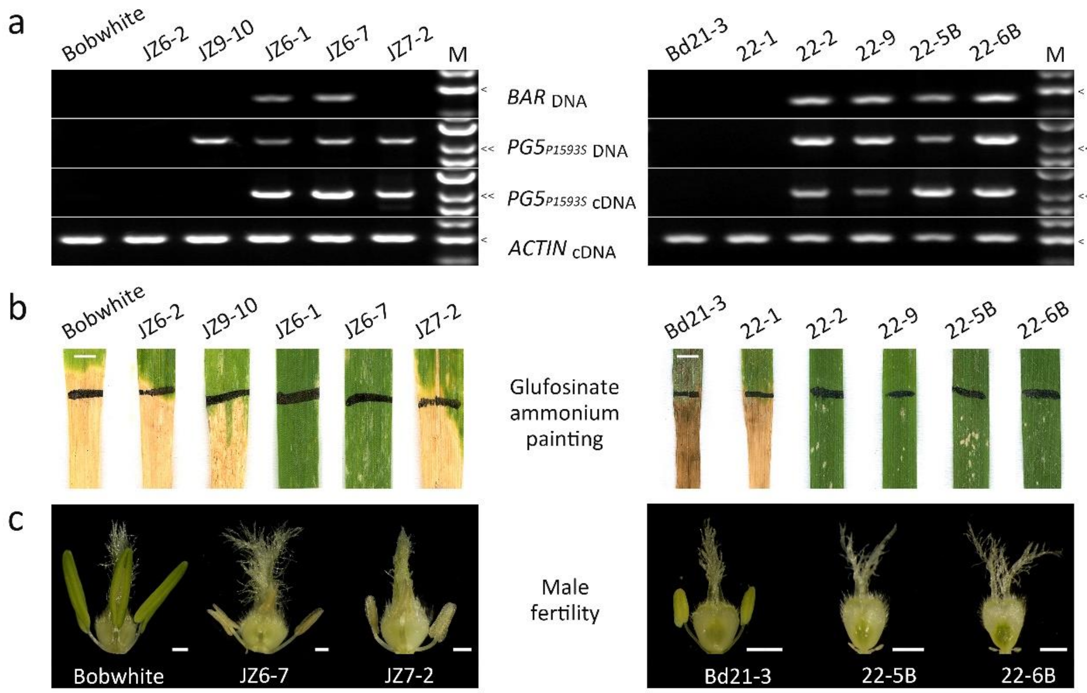


**Supplementary Figure 9. Transformation of wheat (left panel) and *Brachypodium* (right pane) with the *PG5$_{P1593S}$* gene. (a, b)** Co-occurrence of *PG5$_{P1593S}$* DNA, mRNA and herbicide tolerance in transgenic male-sterile (MS) lines and negative phenotypes in two male-fertile (MF) non-transformed controls: 'Bobwhite' wheat and 'Bd21-3' Brachypodium. (**a**) PCR confirmation of transgene integration (*BAR* and *PG5$_{P1593S}$* DNA) and transgene transcription (*PG5$_{P1593S}$* and *ACTIN* cDNA) in $T_0$ plants. A 36-cycle PCR was performed to detect the intergration of *BAR* (P121/P122, 412 bp) and *PG5$_{P1593S}$* (P133/P141, 1,086 bp, or the HT5 marker), and to detect transcripts of *PG5$_{P1593S}$* (P129/P130, 997 bp) and *ACTIN* (P142/P143, 503 bp). (**b**) *BAR*-based bioassay for herbicide tolerance; the herbicide glufosinate ammonium was applied below the black line. (**c**) Male fertility in selected transgenic $T_0$ plants. Other MS transgenics are shown in Fig. 3. PCR primers are described in Methods and Supplementary Data 2. M, the 250bp DNA Ladder ('<' = 0.5kb, '<<' = 1kb). Scale bars = 2.5mm (b) and 1mm (c).

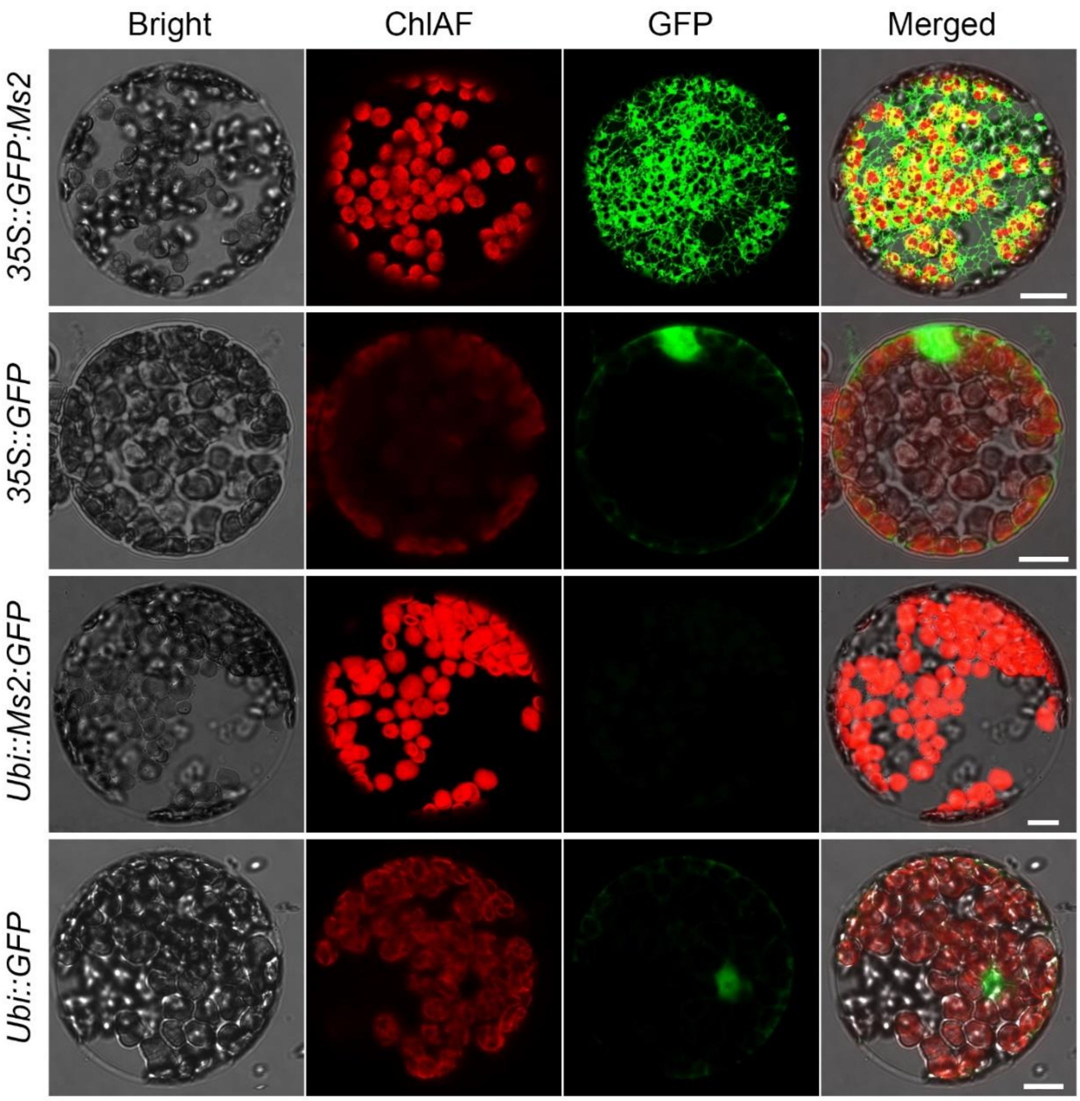


**Supplementary Figure 10. Subcellular location of the GFP:Ms2 protein in mesophyll protoplasts of tobacco.** The GFP signal is shown in green and the chlorophyll autofluorescence (ChlAF) is shown in red. pMDC43 (*35S::GFP*) serves as a control for the *35S::GFP:Ms2* construct (PC983). PC134 (*Ubi::GFP*) serves as a control for the *Ubi::Ms2:GFP* construct (PC972). In the upper row, the localization of GFP:Ms2 (from a gene with a *35S* promoter) appears to be consistent with the rough endoplasmic reticulum. Scale bars = 10µm.

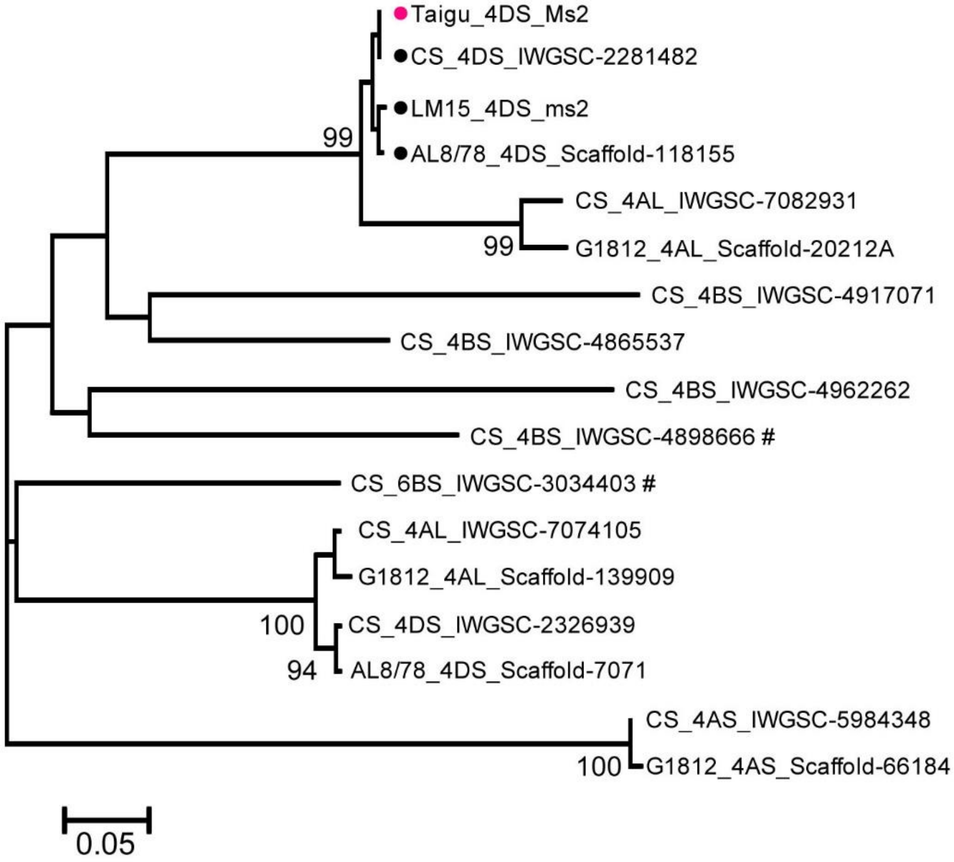


**Supplementary Figure 11. Phylogenetic analysis of the *MS2*-like fragments in Triticeae species.** We used sequence data of accessions of common wheat 'Chinese Spring' (CS) and LM15, and wheat progenitor species: *Aegilops tauschii* accession 'AL8/78' and *Triticum urartu* accession 'G1812'. Phylogenetic analysis was performed on fragments that align with the first five exons of *Ms2*. The program MEGA 6.06[5] was used to align, remove indels and construct a neighbor-joining tree with 1,000 bootstrap iterations. The distance scale bar indicates nucleotide differences per unit length. Bootstrap numbers (>80) are shown at the respective nodes. On the figure, accessions are named as one of the four types (either CS, LM15, Al8/78, or G1812)_the chromosome arm_ and the gene name or accession number from the wheat genome sequence (IWGSC1.0+popseq.28, http://plants.ensembl.org). The dominant *Ms2* (pink circle) and recessive *ms2* (black circle) alleles are shown. Predicted exons, reported in wheat expressed sequence tags, are marked by a pound sign (#). In IWGSC-3034403, the fourth and fifth pseudo-exons are switched; they were switched back for better alignment. In wheat homoeologous group 4, the chromosome arm 4AL is homologous to 4BS and 4DS[6].

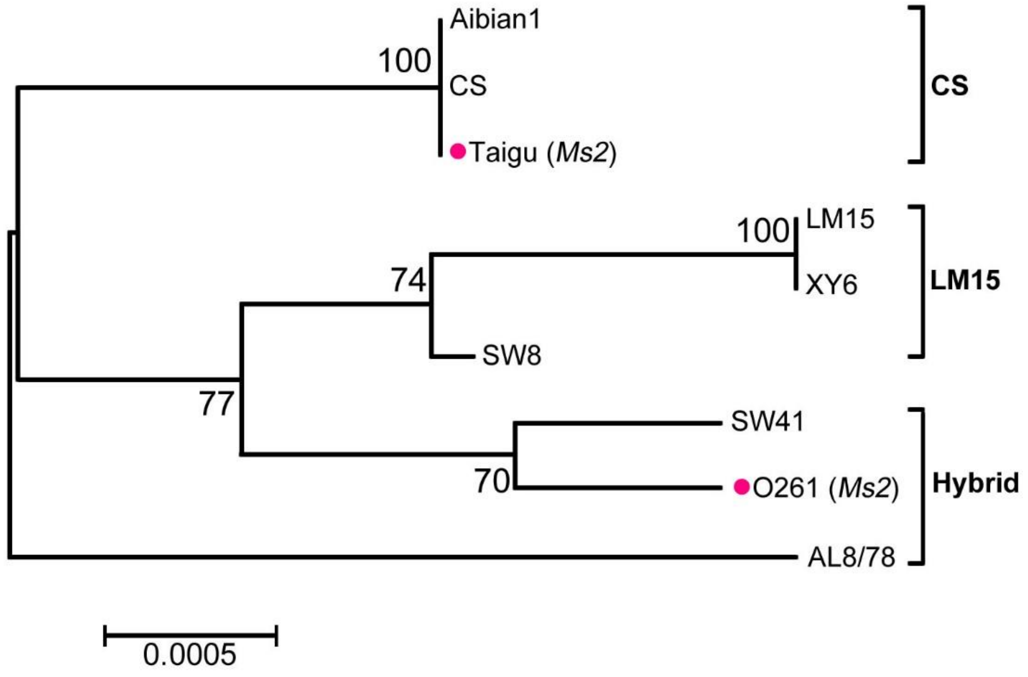


**Supplementary Figure 12. Phylogenetic analysis of the *MS2* full-length genes in wheat 4DS chromosomes.** We selected members of the common wheat 'Chinese Spring' (CS) and LM15 haplogroups, and wheat progenitor species *Aegilops tauschii* accession 'AL8/78'. Phylogenetic analysis was performed using the full-length genomic DNAs (aligned with -805..+507 from the CS genomic sequence, Supplementary Data 3). The program MEGA 6.06[5] was used to align and construct a neighbor-joining tree with 1,000 bootstrap iterations. The distance scale bar indicates nucleotide differences per unit length. Bootstrap numbers (⩾70) are shown in the respective nodes. In this figure, Taigu and O261 are the only male-sterile accessions, each with the dominant *Ms2* (pink circle); the rest of the lines are male-fertile. The *MS2* genes within the CS group are nearly identical; the only difference is in the promoter region of *MS2* between the sterile and fertile lines (Supplementary Fig. 7). The *MS2* gene haplogroups (CS, LM15 and Hybrid on right) are defined in Table 1 and Supplementary Data 8.

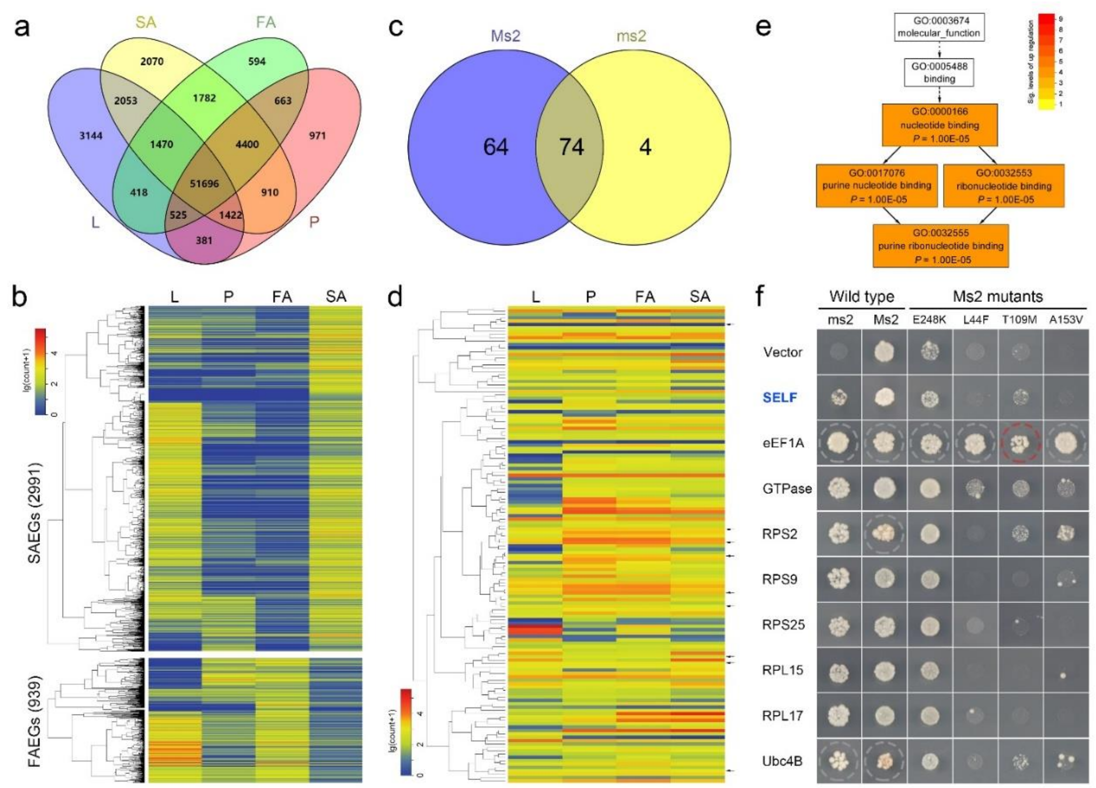


**Supplementary Figure 13.** ***MS2* networks.** (**a**) Venn diagram of 72,499 expressed genes in leaf (L), pistil (P), sterile anther (SA), and fertile anther (FA). (**b**) Heat maps of anther-enriched genes. (**c**) Venn diagram of 142 MS2-interacting proteins. (**d**) Heat map of the 142 MS2-interacting proteins. (**e**) GO analysis of the 142 MS2-interacting proteins. The color of a node matches the significance (sig.) bar and a *P*-value was included for the significant node. (**f**) Protein interactions between MS2 (bait, on top) and representative full-length proteins (prey, on left). Yeast colonies (50-fold dilution) were grown on the triple dropout medium. Dashed circles indicate yeast growth on the quadruple dropout medium, and a dashed red circle indicates weak growth. All tested Ms2 variants confers male-fertility. Vector, the prey vector; SELF, the same protein used as the bait; RPSs and RPLs, ribosomal proteins; Ubc4B, ubiquitin-conjugating enzyme.

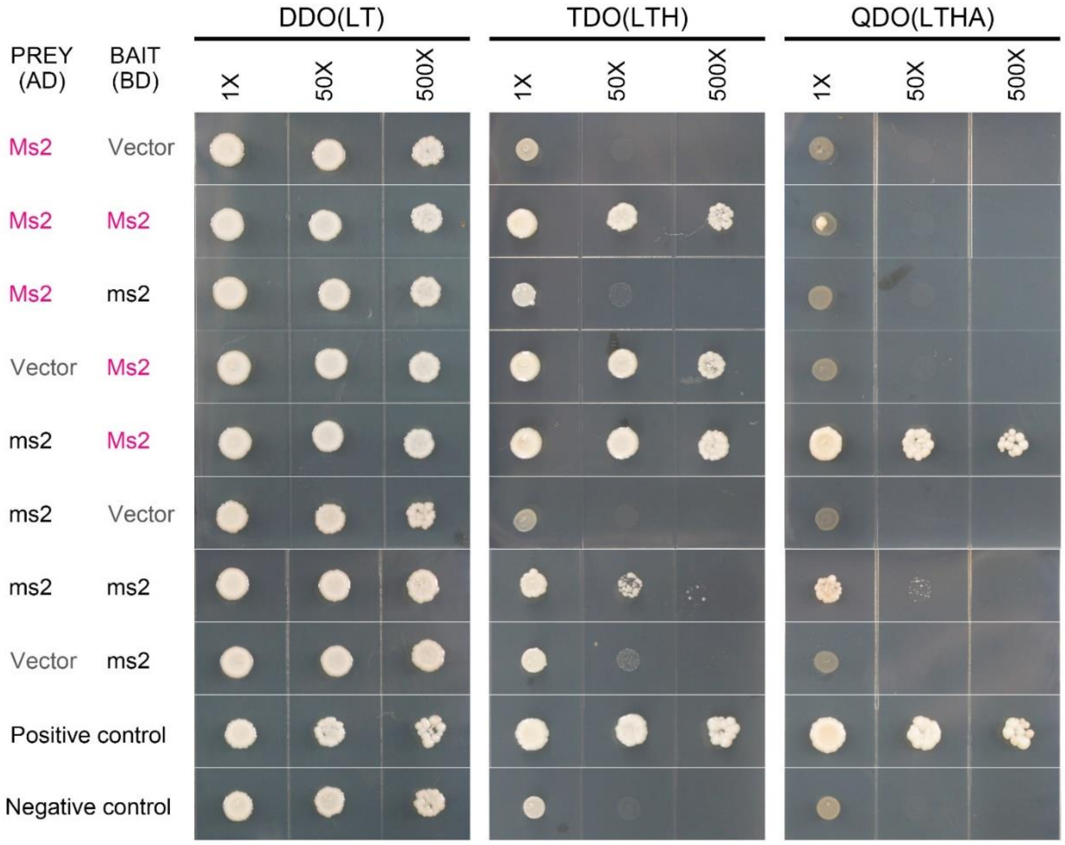


**Supplementary Figure 14. Yeast two-hybrid analysis of the MS2 to ms2 proteins.** Ms2 is from $PG5_{P1593S}$, and ms2 is from $PG5_{P1076F}$. Yeast two-hybrid analysis was performed on a Gal4 system including the AD vector (prey) and BD vector (bait). Successful co-transformation of AD and BD plasmids was verified by yeast growth in the DDO medium, whereas autoactivation or interaction was tested in TDO and QDO media. The yeast colonies were progressively diluted from the original suspension (1x), 1:50-fold dilution (50x), and 1:500-fold dilution (500x). The Y2H assay included the positive control (BD-wXb12 and AD-wXb12IP2) and negative control (BD-wXb12 and AD-wRAR1)[7]. Ms2 and ms2 had no autoactivity on the TDO and QDO media except for Ms2 as a bait on TDO. Ms2 (bait) also interacted with ms2 (prey) on QDO; ms2 interacted with itself on TDO. Potentially, MS2 proteins may form a dimer or multimer.

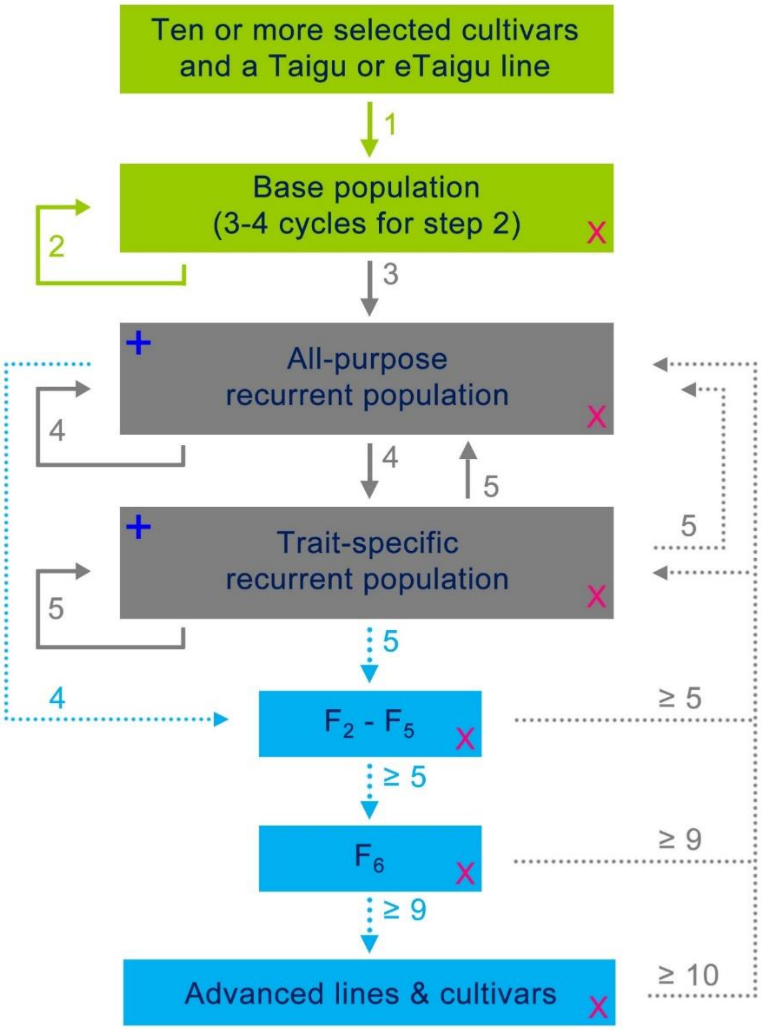


**Supplementary Figure 15.** ***Ms2*-based recurrent selection in wheat.** This procedure, adapted from previous studies[8-11], includes the use of either wild-type Taigu or 'engineered Taigu' (eTaigu) lines that have a *Ms2* gene. The procedure includes the development of an initial "base" population (green), the development and maintenance of recurrent populations (gray), and the release of advanced lines and cultivars (light blue). The number of steps in the simplest path is indicated in the center. The number of steps in more complicated paths is marked by a 'greater than or equal to' sign (≥). A solid step line indicates bulk seeds from selected male sterile (MS) plants and a dashed step line indicates seeds from selected male fertile (MF) plants. Seeds of MF plants can be used to maintain the recurrent populations (dashed lines in gray) and to develop advanced lines and cultivars (dashed lines in light blue). To enrich the genetic variability in recurrent populations, new genotypes/lines can be added into the recurrent populations, as marked by a plus sign (+). During the course of recurrent selection and progeny tests, undesirable plants/lines will be removed, as labeled by an x-shaped cross.

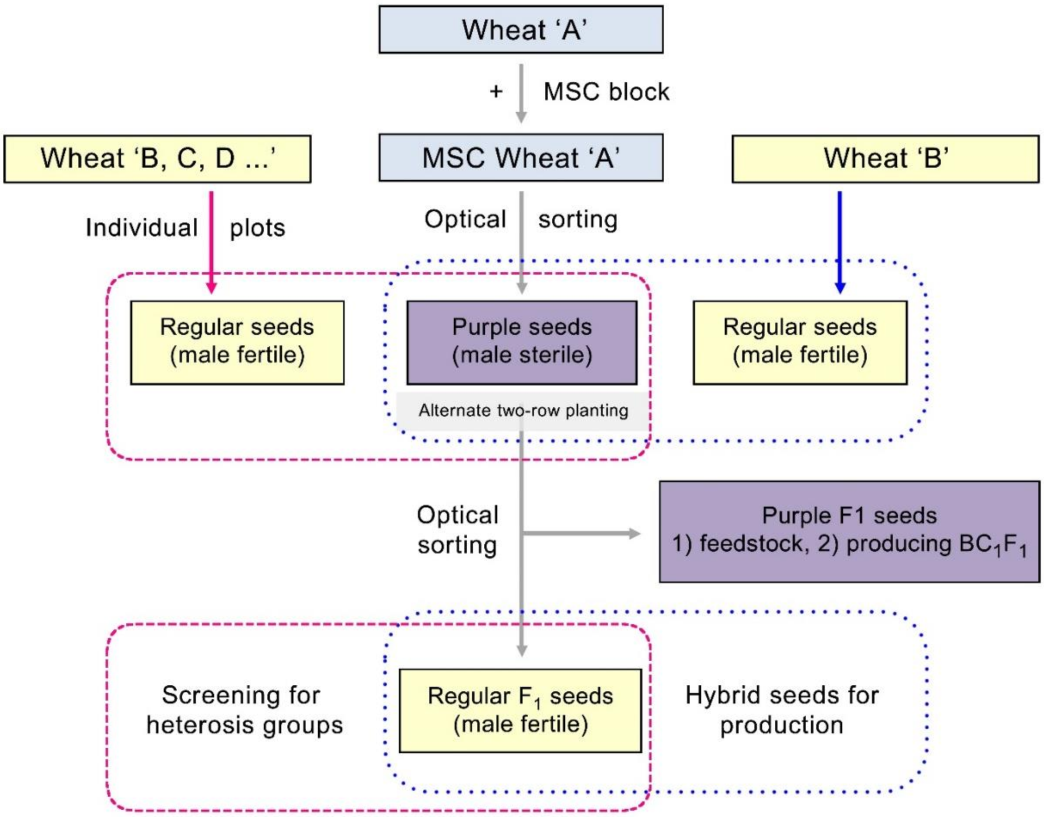


**Supplementary Figure 16. A schematic representation of a system for producing hybrid wheat seed using *Ms2*.** The system is based on the use of Ms2 color wheat (MSC). The MSC cassette is composed of two linked genes: *Ms2* for male-sterility and an aleurone-specific gene for pigmented grain. Wheat 'A' is first transformed to MSC wheat, amplified, and then planted in alternate rows. Within individual plots, pollen donors of a specific cultivar are planted in each row adjacent to the MSC wheat. All seed in the MSC rows will be a $F_1$ hybrid, and will be planted in the field and examined for desirable traits. $F_1$s of each specific cross between one cultivar and an MSC line will be evaluated in replicated plots to identify specific heterotic groups of interest (dashed pink lines). Specific MSC wheat and its heterotic partner will be used to generate a large amount of $F_1$ seeds (dashed blue lines), and their hybrid vigor will be determined on the population level. The most promising heterotic groups from specific cultivars will be scaled up for hybrid seed production. The system also generates MSC-type $F_1$ hybrids, which can be used directly as feedstocks or used to prepare $BC_1F_1$ populations. During the process, high throughput optical sorting is used to separate pigmented seeds from wild type seeds.

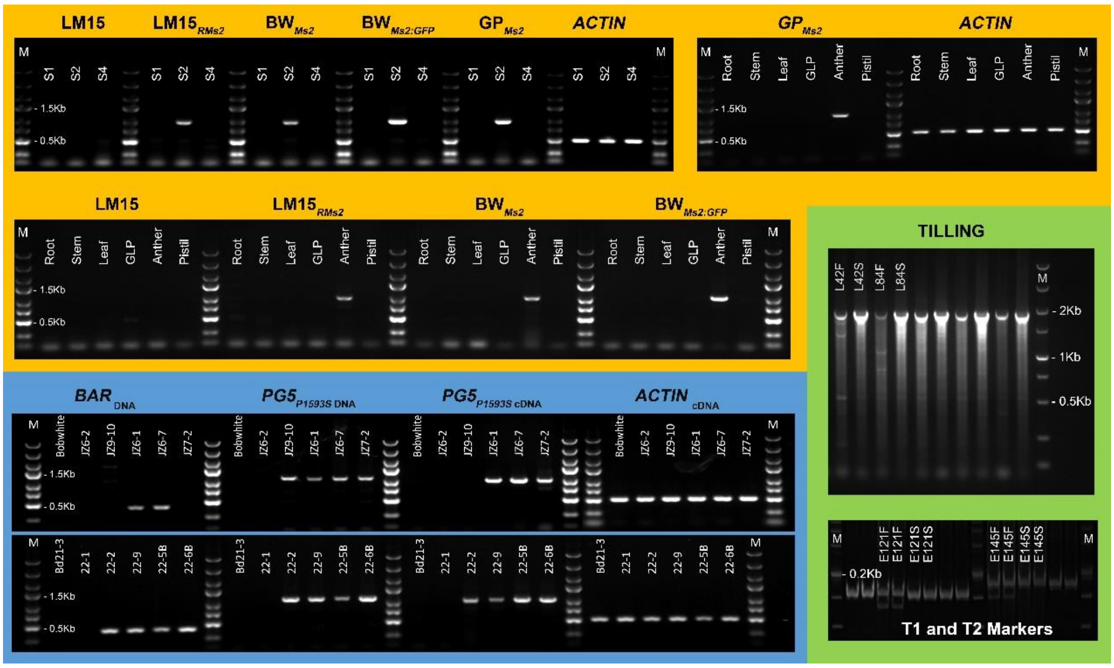


**Supplementary Figure 17. Uncropped images with molecular markers.** Gels with an orange background have treatments in Fig. 4a,b, those with a green background have treatments in Supplementary Fig. 8b,d, and those with a blue background have treatments shown in Supplementary Fig. 9a. M, the 250bp DNA Ladder (those with an orange or blue background), the DS5000 DNA Ladder (TILLING with a green background), and the DM500 DNA Ladder (T1 and T2 Markers with a green background).

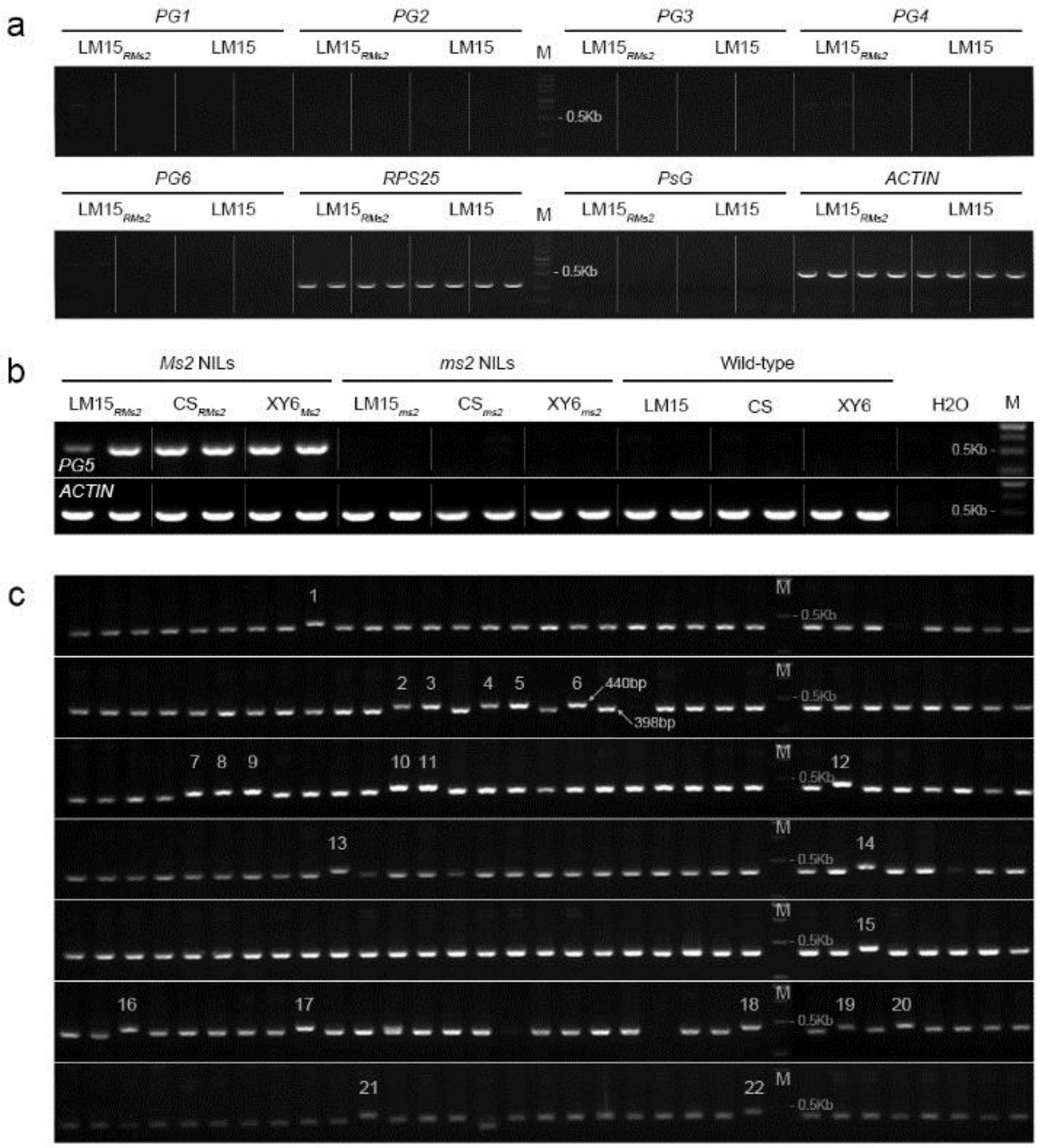


**Supplementary Figure 18. Uncropped images with molecular markers.** (**a**) The expression of seven potential genes in the *Xsdauw24-Xsdauw32* interval. (**b**) The expression of *PG5* in the *Ms2* near-isogenic lines (NIL) and the wild-type lines. The top panel is for *PG5* (P115/P136, 504 bp), and the lower panel is for *ACTIN* control (P142/P143, 503bp). (**c**) A CAPS marker was used to differentiate the *PG5* cDNA clones (P104/P100, 440 bp). M, the DS5000 DNA Ladder (Dongsheng). H2O, negative control for RT-PCR.

## Supplementary Tables

**Supplementary Table 1. Plant materials used in the current study [a]**

| ID/Accession No. | Abbreviation | Species (pedigree) | Genome |
|---|---|---|---|
| AL8/78 | - | *Aegilops tauschii* Coss. | DD |
| CIae 9 | - | *Aegilops tauschii* Coss. | DD |
| PI 560536 | - | *Aegilops tauschii* Coss. | DD |
| Langdon / CItr 13165 | LDN | *Triticum turgidum* L. | AABB |
| LDN-DIC 2B(521) | $LDN_{DIC521\text{-}2B}$ [b] | *Triticum turgidum L.* | AABB |
| SW7 | SW7 [c] | Synthetic hexaploid wheat | AABBDD |
| SW8 | SW8 [c] | Synthetic hexaploid wheat | AABBDD |
| SW41 | SW41 [c] | Synthetic hexaploid wheat | AABBDD |
| Chinese Spring+*RMs2* | $CS_{RMs2}$ | *Triticum aestivum* L. | AABBDD |
| Lumai 15+*RMs2* | $LM15_{RMs2}$ | *Triticum aestivum* L. | AABBDD |
| Xiaoyan 6+*Ms2* | $XY6_{Ms2}$ | *Triticum aestivum* L. | AABBDD |
| $BC_1F_1$ population A | popA | ($XY6_{Ms2}$/2*SW41) | AABBDD |
| $BC_1F_1$ population D | popD | ($XY6_{Ms2}$/2*SW7) | AABBDD |
| $BC_1F_1$ population E | popE | ($LM15_{RMs2}$/2*SW41) | AABBDD |
| $BC_1F_1$ population F | popF | ($CS_{RMs2}$/2*SW41) | AABBDD |
| Chinese Spring | CS | *Triticum aestivum* L. | AABBDD |
| Lumai 15 | LM15 | *Triticum aestivum* L. | AABBDD |
| Xiaoyan 6 | XY6 | *Triticum aestivum* L. | AABBDD |
| Bobwhite | BW | *Triticum aestivum* L. | AABBDD |
| Golden Promise / PI 343079 | GP | *Hordeum vulgare* L. | HH |
| Bd21 / PI 254867 | BD | *Brachypodium distachyon* L. | - |
| - | - | *Nicotiana benthamiana* Domin | - |

[a]Wheat cultivars/lines used for haplotype analysis and genetic diversity are listed in Supplementary Data 8. [b]The 2B chromosome in Langdon was replaced by the one from wild emmer wheat 'PI 481521' (*Triticum turgidum* ssp. *dicoccoides*). [c]Pedigrees of SW7, SW8, and SW41 are Langdon/*Ae. tauschii* CIae22, Langdon/*Ae. tauschii* CIae25, and Langdon/*Ae. tauschii* RL5570, respectively. '-' not applicable or not accessible.

**Supplementary Table 2. BAC library of LM15$_{RMs2}$**

| Batch Codes | Plate Quantity | Empty Rate (%) | Insert Size (kb) [a] | Clone Quantity | Genome Coverage |
|---|---|---|---|---|---|
| A | 1112 | 0 | 118 | 427,008 | 2.97 |
| B | 330 | 0.81 | 132.2 | 126,720 | 0.98 |
| C | 255 | 2.59 | 147.6 | 97,920 | 0.83 |
| D | 142 | 0 | 102.1 | 54,528 | 0.33 |
| Total | 1839 | 0.5 | 123.32 | 706,176 | 5.1 |

[a]Insert size was averaged among non-empty clones.

**Supplementary Table 3. Plasmid constructs used in this study**

| PC NO. | Expression Cassette [a] | Target Gene [b] | GenBank Acc. [d] | Vector Backbone |
|---|---|---|---|---|
| pMDC43 | *35S::GFP6* | *GFP6* | - | pMDC43 |
| PC134 | *Ubi::GFP* | *GFP* | - | pPZP200 |
| PC970 | $Ubi::PG5_{P1593S}$ | $PG5_{P1593S} = Ms2$ | KX533929 | pCAMBia1300 |
| PC971 | $Ubi::PG5_{P1076F}$ | $PG5_{P1076F} = ms2$ | KX533930 | pCAMBia1300 |
| PC972 | $Ubi::PG5_{P1593S}:GFP$ | $PG5_{P1593S} = Ms2$ | KX533929 | pCAMBia1300 |
| PC973 | $NP1::PG5_{P1593S}:GFP$ | $PG5_{P1593S} = Ms2$ [c] | KX585234 | pCAMBia1300 |
| PC976 | $NP1::PG5_{P1593S}$ | $PG5_{P1593S} = Ms2$ [c] | KX585234 | pCAMBia1300 |
| PC983 | *35S::GFP:Ms2* | *Ms2* | KX533929 | pMDC43 |
| PC322 | *ADH1::AD:wRAR1* | *wRAR1* | EF202841 | pGADT7 |
| PC324 | *ADH1::AD:wXb12IP2* | *wXb12IP2* | JX424313 | pGADT7 |
| PC332 | *ADH1::BD:wXb12* | *wXb12* | JX424306 | pGBKT7 |
| PC986 | *ADH1::AD:Ms2* | *Ms2* | KX533929 | pGADT7 |
| PC987 | *ADH1::BD:Ms2* | *Ms2* | KX533929 | pGBKT7 |
| PC988 | *ADH1::AD:ms2* | *ms2* | KX533930 | pGADT7 |
| PC989 | *ADH1::BD:ms2* | *ms2* | KX533930 | pGBKT7 |
| PC1034 | $ADH1::AD:Ms2^{E248K}$ | $Ms2^{E248K}$ | - | pGADT7 |
| PC1035 | $ADH1::BD:Ms2^{E248K}$ | $Ms2^{E248K}$ | - | pGBKT7 |
| PC1036 | $ADH1::AD:Ms2^{L44F}$ | $Ms2^{L44F}$ | - | pGADT7 |
| PC1037 | $ADH1::BD:Ms2^{L44F}$ | $Ms2^{L44F}$ | - | pGBKT7 |
| PC1040 | $ADH1::AD:Ms2^{T109M}$ | $Ms2^{T109M}$ | - | pGADT7 |
| PC1041 | $ADH1::BD:Ms2^{T109M}$ | $Ms2^{T109M}$ | - | pGBKT7 |
| PC1044 | $ADH1::AD:Ms2^{A153V}$ | $Ms2^{A153V}$ | - | pGADT7 |
| PC1045 | $ADH1::BD:Ms2^{A153V}$ | $Ms2^{A153V}$ | - | pGBKT7 |
| PC991 | *ADH1::AD:eEF1A* | *eEF1A* | KX533924 | pGADT7 |
| PC995 | *ADH1::AD:GTPase* | *GTPase* | KX533926 | pGADT7 |
| PC1016 | *ADH1::AD:RPS2* | *RPS2* | KX533927 | pGADT7 |
| PC1018 | *ADH1::AD:RPS9* | *RPS9* | HX055302 | pGADT7 |
| PC1020 | *ADH1::AD:RPS25* | *RPS25* | HX019260 | pGADT7 |
| PC1022 | *ADH1::AD:RPL15* | *RPL15* | HX250311 | pGADT7 |
| PC1024 | *ADH1::AD:RPL17* | *RPL17* | HX188548 | pGADT7 |
| PC1032 | *ADH1::AD:Ubc4B* | *Ubc4B* | HX066634 | pGADT7 |

[a]*Ubi*, the maize ubiquitin promoter[12]; *NP1*, a 5,578-bp native promoter upstream of the start codon of the $PG5_{P1593S}$ gene; 35S, 2× 35S promoter[13]; *ADH1*, a truncated promoter of the *alcohol dehydrogenase 1* gene from *Saccharomyces cerevisiae*[14].
[b]Most are cDNA copies. $PG5_{P1593S} = Ms2$, $PG5_{P1076F} = ms2$, and both from $LM15_{RMs2}$; $Ms2^{L44F}$, $Ms2^{T109M}$, $Ms2^{A153V}$, and $Ms2^{E248K}$, single-base mutations of the wild-type *Ms2* gene; *GFP*, green fluorescent protein gene[15]; *GFP6*, a modified *GFP* gene[16];

*eEF1A*, eukaryotic elongation factor 1A; *GTPase*, GTP-binding protein; *RPSs* and *RPLs*, ribosomal proteins; *Ubc4B*, ubiquitin-conjugating enzyme; *wRAR1*, *wXb12*, and *wXb12IP2*, control genes used for yeast-two analysis[7]. [c]genomic DNA. [d]GenBank accessions of the target genes. '-' not applicable or not accessible.

**Supplementary Table 4. Homologous DNA of the *MS2* gene in Triticeae**

| Species/Cultivars | Contig or Scaffold [a] | Chr. | Homologous Exons [b] | Chromosome Location [c] |
|---|---|---|---|---|
| *Ta*/CS | 7082931 | 4AL | Ex1-8 | 4A:171806160:171813880 [j] |
| *Tu*/G1812 | 20212A | 4AL | Ex1-8 | 4AL_scaff_20212:158766:161615 [k] |
| *Ta*/CS | 7074105 | 4AL | Ex1-8; Ex7\|6 [f] | 4A:170957681:170967985 |
| *Tu*/G1812 | 139909 | 4AL | Ex1-8; Ex7\|6 [f] | 4AL_scaff_139909:8409:18024 |
| *Ta*/CS | 4898666 [d] | 4BS | Ex1-6; Ex7+8 [g] | 4B:1148925:1155030 |
| *Ta*/CS | 4962262 | 4BS | Ex1-5; Ex7+8 [g] | 4B:1473786:1479383 |
| *Ta*/CS | 2281482 | 4DS | Ex1-8 (*ms2*) | 4DS_scaff_2281482:24011:31879 |
| *Ae*/AL8/78 | 118155 | 4DS | Ex1-8 (*ms2*) | 4DS_scaff_118155:33768:42646 |
| *Ta*/CS | 2326939 | 4DS | Ex1-8; Ex7\|6 [f] | 4DS_scaff_2326939:2426:9990 |
| *Ae*/AL8/78 | 7071 | 4DS | Ex1-8; Ex7\|6 [f] | 4DS_scaff_7071:68422:76053 |
| *Ta*/CS | 3034403 [e] | 6BS | Ex1-5; Ex5\|4 [f] | 6B:98153352:98157312 |
| *Ta*/CS | 5984348 | 4AS | Ex1-5, Ex4 [h]; Ex7 [i] | 4AS_scaff_5984348:5509:12263 |
| *Tu*/G1812 | 66184 | 4AS | Ex1-5, Ex4 [h]; Ex7 [i] | 4AS_scaff_66184:12694:21363 |
| *Ta*/CS | 7148227 | 4AL | Ex1; Ex2 [i] | 4A:172121151:172121574 |
| *Tu*/G1812 | 20212B | 4AL | Ex1; Ex2 [i] | 4AL_scaff_20212:134552:134789 |
| *Ta*/CS | 4865537 | 4BS | Ex1-3, 5-6 | 4BS_scaff_4865537:5609:11586 |
| *Ta*/CS | 4917071 | 4BS | Ex1-2, 4-5, 7, 8 | 4B:820974:824787 |
| *Ta*/CS | 4871850 | 4BS | Ex1 | 4B:2203566:2203808 |
| *Ta*/CS | 4945153 | 4BS | Ex4 | 4B:90873:90954 |
| *Ta*/CS | 2998791 | 6BS | Ex5 | 6B:85762514:85762595 |
| *Ta*/CS | 4928922 | 4BS | Ex6 | 4B:176356757:176356887 |
| *Ta*/CS | 4860697 | 4BS | Ex3, 6 | 4B:412957:414442 |
| *Ta*/CS | 4951082 | 4BS | Ex3, 6 | 4B:1613684:1614023 |
| *Ta*/CS | 4882842 | 4BS | Ex7+8 [g] | 4B:1224732:1224857 |
| *Ta*/CS | 4910553 | 4BS | Ex7+8 [g] | 4BS_scaff_4910553:2510:2622 |

[a]A representative contig/scaffold is listed. [b]The order of exons was based on the dominant *Ms2* gene (GenBanck: KX585234). [c]Chromosome of 'Chinese Spring' (IWGSC1.0+popseq.28, http://plants.ensembl.org). [d]Shares five exons with wheat EST AK331503. [e]Shares two exons with wheat EST BE500370. [f]Predicted exons inverted in order. [g]Predicted exons mapped to the same region. [h]Exon duplicated. [i]Predicted exon partially retained. [j]Alternative locations (4A:171550005:171557187 and 4A:172222864:172224346). [k]Alternative scaffolds (scaffold101798:1:2597 and scaffold139909:61872:70085).

**Note:** *Ae*/AL8/78, *Aegilops tauschii* accession 'AL8/78'; *Ta*/CS, *Triticum aestivum* cultivar Chinese Spring; *Tu*/G1812, *Triticum urartu* accession 'G1812'. Fonts in gray indicate a predicted chromosome arm and its location in a sequence scaffold.

**Supplementary Table 5. Counts of paired-end (PE) reads**

| Samples | Trials | Clean Pairs | Clean Bases (bp) | Read Length (bp) | GC Content (%) |
|---|---|---|---|---|---|
| Fertile anther (FA) | 1 | 37,863,983 | 9,465,995,750 | 125 | 54.40% |
| | 2 | 44,837,668 | 11,209,417,000 | 125 | 54.80% |
| | 3 | 38,790,150 | 9,697,537,500 | 125 | 54.00% |
| Sterile anther (SA) | 1 | 63,136,384 | 15,784,096,000 | 125 | 55.20% |
| | 2 | 38,953,021 | 9,738,255,250 | 125 | 56.30% |
| | 3 | 41,563,567 | 10,390,891,750 | 125 | 54.90% |
| Leaf (L) | 1 | 35,551,128 | 8,887,782,000 | 125 | 54.30% |
| | 2 | 39,012,451 | 9,753,112,750 | 125 | 53.90% |
| | 3 | 36,765,025 | 9,191,256,250 | 125 | 54.90% |
| Pistil (P) | 1 | 33,563,458 | 8,390,864,500 | 125 | 54.70% |
| | 2 | 38,232,211 | 9,558,052,750 | 125 | 55.10% |
| | 3 | 35,980,271 | 8,995,067,750 | 125 | 54.30% |
| Total | 12 | 484,249,317 | 121,062,329,250 | 125 | 54.76% |

**Supplementary Table 6. Counts of mapped paired-end (PE) reads**

| Samples | Trials | Unique Mapped Reads | | Multiple Mapped Reads | | Unmapped Reads | |
|---|---|---|---|---|---|---|---|
| | | Clean Pairs | Percentage | Clean Pairs | Percentage | Clean Pairs | Percentage |
| Fertile anther (FA) | 1 | 29,162,254 | 77.02% | 3,692,938 | 9.80% | 5,008,791 | 13.23% |
| | 2 | 34,806,413 | 77.63% | 4,171,441 | 9.30% | 5,859,814 | 13.07% |
| | 3 | 29,829,956 | 76.90% | 3,787,733 | 9.80% | 5,172,461 | 13.33% |
| Sterile anther (SA) | 1 | 48,501,465 | 76.82% | 5,567,749 | 8.80% | 9,067,170 | 14.36% |
| | 2 | 28,733,927 | 73.77% | 3,444,548 | 8.80% | 6,774,546 | 17.39% |
| | 3 | 31,655,229 | 76.16% | 3,604,604 | 8.70% | 6,303,734 | 15.17% |
| Leaf (L) | 1 | 21,875,846 | 61.53% | 3,589,986 | 10.10% | 10,085,296 | 28.37% |
| | 2 | 23,131,090 | 59.29% | 4,057,438 | 10.40% | 11,823,923 | 30.31% |
| | 3 | 24,059,675 | 65.44% | 3,593,468 | 9.80% | 9,111,882 | 24.78% |
| Pistil (P) | 1 | 25,488,877 | 75.94% | 3,461,341 | 10.30% | 4,613,240 | 13.74% |
| | 2 | 29,098,662 | 76.11% | 4,075,446 | 10.70% | 5,058,103 | 13.23% |
| | 3 | 27,348,856 | 76.01% | 3,744,926 | 10.40% | 4,886,489 | 13.58% |
| Total | 12 | 353,692,250 | 73.04% | 46,791,618 | 9.66% | 83,765,449 | 17.30% |

**Supplementary Table 7. Characterization of the normalized and cleaned counts of RNA-seq**

| Samples | Expressed Genes (%) [ab] | Gene Expression Level (count value) [a] | | | | | Expressed Genes (%) [bc] |
|---|---|---|---|---|---|---|---|
| | | Mean (SE [d]) | Median | 5th | 10th | 50th | |
| Fertile anther (FA) | 69,459 (62%) | 275 (5) | 47 | 0.28 | 0.80 | 47 | 61,548 (55%) |
| Sterile anther (SA) | 73,300 (65%) | 309 (7) | 44 | 0.43 | 1.04 | 44 | 65,803 (58%) |
| Leaf (L) | 65,591 (58%) | 469 (19) | 58 | 0.64 | 1.55 | 58 | 61,109 (54%) |
| Pistil (P) | 68,522 (61%) | 247 (3) | 49 | 0.28 | 0.83 | 49 | 60,968 (54%) |
| All [a] | 79,153 (70%) | 323 (5) | 49 | 0.52 | **1.06** [e] | 49 | na |
| All [c] | na | 358 (6) | 67 | 1.87 | **3.29** | 67 | **72,499 (64%)** |

[a]Results based on the normalized counts before removing false positives. [b]At least one nonzero count value (count > 0) in each of three trials. [c]Results based on the corrected counts after removing false positives. [d]Standard error (SE) of the mean. [e]A count cutoff value was determined as the 10th percentile of expression levels[17]. Percentages were calculated based on 112,495 genes. na, not applicable.

## Supplementary References


1. Deng, J. & Gao, Z. Discovery and determination of a dominant male-sterile gene and its importance in genetics and wheat breeding. *Scientia Sinica (Series B)* **25**, 508-516 (1982).
2. Zhang, Y., Deng, J. & Ji, F. A study for stability of male sterility of Taigu genic male sterile wheat. *Shanxi Agricultural Sciences* **6**, 5-8 (in Chinese) (1987).
3. Wang, S. *et al.* Characterization of polyploid wheat genomic diversity using a high-density 90 000 single nucleotide polymorphism array. *Plant Biotechnology J.* **12**, 787–796 (2014).
4. Luo, M.-C. *et al.* A 4-gigabase physical map unlocks the structure and evolution of the complex genome of *Aegilops tauschii*, the wheat D-genome progenitor. *Proc. Natl Acad. Sci. USA* **110**, 7940-7945 (2013).
5. Tamura, K., Stecher, G., Peterson, D., Filipski, A. & Kumar, S. MEGA6: Molecular evolutionary genetics analysis version 6.0. *Mol. Biol. Evol.* **30**, 2725-2729 (2013).
6. Mickelson-Young, L., Endo, T. R. & Gill, B. S. A cytogenetic ladder-map of the wheat homoeologous group-4 chromosomes. *Theor. Appl. Genet.* **90**, 1007-1011 (1995).
7. Cantu, D. *et al.* Comparative analysis of protein-protein interactions in the defense response of rice and wheat. *BMC Genomics* **14**, 166 (2013).
8. Gao, Q. *et al.* Method and technique of population improving for quality characteristics. *Journal of Triticeae Crops* **25**, 19-23 (in Chinese) (2005).
9. Yang, L., Wang, S. & Liu, B. Building excellence breeding technology and system using dwarfing sterile wheat. *Review of China Agricultural Science and Technology* **6**, 8-10 (in Chinese) (2004).
10. Yang, L. *et al.* Dwarf male-sterile wheat: A revolutionary breeding approach to wheat. in *Induced Plant Mutations In The Genomics Era* (ed. Shu, Q. Y.) 370-372 (Food and Agriculture Organization of the United Nations, Rome, Italy, 2009).
11. Sorrells, M. E. & Fritz, S. E. Application of a dominant male-sterile allele to the improvement of self-pollinated crops. *Crop Sci.* **22**, 1033-1035 (1982).
12. Christensen, A., Sharrock, R. & Quail, P. Maize polyubiquitin genes: structure, thermal perturbation of expression and transcript splicing, and promoter activity following transfer to protoplasts by electroporation. *Plant Mol. Biol.* **18**, 675-689 (1992).
13. Kay, R., Chan, A., Daly, M. & Mcpherson, J. Duplication of CaMV 35S promoter sequences creates a strong enhancer for plant genes. *Science* **236**, 1299-1302 (1987).
14. Louvet, O., Doignon, F. & Crouze, M. Stable dna-binding yeast vector allowing high-bait expression for use in the two-hybrid system. *BioTechniques* **23**, 816-820 (1997).
15. Chiu, W.-l. *et al.* Engineered GFP as a vital reporter in plants. *Current biol.* **6**, 325-330 (1996).
16. Schuldt, A. J. *et al.* Miranda mediates asymmetric protein and RNA localization in the developing nervous system. *Genes Dev.* **12**, 1847-1857 (1998).

17. IWGSC. A chromosome-based draft sequence of the hexaploid bread wheat (*Triticum aestivum*) genome. *Science* **345**, 1251788 (2014).
18. Du, Z., Zhou, X., Ling, Y., Zhang, Z. & Su, Z. agriGO: a GO analysis toolkit for the agricultural community. *Nucleic Acids Res.* **38**, W64-W70 (2010).
19. Benjamini, Y. & Yekutieli, D. The control of the false discovery rate in multiple testing under dependency. *Ann. Stat.* **29**, 1165-1188 (2001).
